# Superior Mesenteric Artery Syndrome Masquerading As Irritable Bowel Syndrome: A Case Report

**DOI:** 10.7759/cureus.43623

**Published:** 2023-08-17

**Authors:** Andrew Lurie, Lohitha Guntupalli, Veenah Stoll, Anthony Moon

**Affiliations:** 1 Internal Medicine, Nova Southeastern University Dr. Kiran C. Patel College Of Osteopathic Medicine, Clearwater, USA; 2 Medicine, Nova Southeastern University Dr. Kiran C. Patel College Of Osteopathic Medicine, Clearwater, USA; 3 Family Medicine, Lakeland Regional Health Medical Center, Lakeland, USA

**Keywords:** cast syndrome, wilkie syndrome, intractable abdominal pain, s: irritable bowel syndrome, small-bowel obstruction, s: superior mesenteric artery syndrome

## Abstract

Superior Mesenteric Artery (SMA) syndrome is a rare upper gastrointestinal problem characterized by narrowing the angle between the aorta and SMA. While the syndrome describes small bowel obstruction as a result of compression at the proximal duodenum, the nonspecific abdominal findings often obscure this diagnosis initially. We present a case of a 21-year-old female with persistent upper abdominal pain of unclear etiology, which was later diagnosed as SMA Syndrome and referred for surgical management. Nonspecific abdominal symptoms in a young female with weight loss and ambiguous laboratory and physical exam findings should increase the index of suspicion for SMA syndrome to mitigate the worsening of serious complications with early treatment.

## Introduction

The superior mesenteric artery (SMA) is a major branch of the abdominal aorta which supplies blood to the pancreas, parts of the small intestine, and the large intestine. It arises just inferior to the origin of the celiac trunk at the L1 or L2 vertebra level. SMA syndrome is the occlusion of the duodenum by a constriction between the abdominal aorta and the SMA. The most common presentation occurs due to a decrease in the retroperitoneal fat pad and connective tissue covering the SMA with a subsequent narrowing of the angle between the SMA and aorta [1,2]. The aortomesenteric angle (AO) is normally between 38 to 65 degrees on CT angiography. An angle of less than 25 degrees with a distance of less than ten millimeters can cause compression and obstruction of the third part of the duodenum [2]. This is commonly seen in patients who have undergone significant weight loss. SMA Syndrome has been reported with the greatest frequency in young females with reduced BMI. However, additional risk factors can include trauma, malabsorption, anorexia, scoliosis, or increased lumbar lordosis and adhesions [1,2]. 

A patient may present acutely with a small bowel obstruction and dilation of the stomach. Alternatively, they may also present with a chronic picture more consistent with a cyclical pattern of weight loss and decreased appetite, leading to further weight loss and exacerbation of the reduced AO angle [2]. Signs and symptoms, including long-standing nausea, vomiting, and postprandial, intermittent, or chronic abdominal pain, can be mischaracterized for other conditions. Peptic ulcer disease and irritable bowel syndrome present similarly with postprandial pain, early satiety, weight loss, and abdominal distention. SMA Syndrome can be a diagnosis of exclusion, though providers should have a high clinical index of suspicion based on demographics and presenting symptomatology so as not to delay confirmatory imaging and to prevent significant complications.

## Case presentation

A 21-year-old female with a BMI of 19.85 and a history of anemia, GERD, and multiple food sensitivities had been reporting chronic postprandial, sharp, intermittent upper abdominal pain for the past two years. The patient previously underwent a workup including esophagogastroduodenoscopy and colonoscopy, both with biopsy, which were all negative. Based on these results, she was diagnosed with irritable bowel syndrome and managed with hyoscyamine, which initially provided some relief.

Due to the persistence of these symptoms and a continued weight loss of nine pounds, the patient presented to our primary care office for more detailed diagnostic examinations as a new patient. The patient reported worsening, consistent sharp, stabbing epigastric, right upper quadrant, and right lower quadrant pain that alleviated with defecation. A review of systems revealed feeling hot, flushed, and lightheaded soon after eating food, without any complaints of fevers, constipation, or diarrhea. 

The patient’s vitals on physical examination revealed tachycardia at 112 beats per minute with a regular rhythm (Table 1). An abdominal exam revealed no distention, masses, or tenderness to palpation. Serum laboratory studies were nonspecific (Table 2).

**Table 1:** Vital signs significant for tachycardia and low-normal BMI BMI: Body Mass Index

	Value	Reference Range
Blood Pressure	116/68 mmHg	<120/ <80 mmHg
Pulse	112 beats per minute	60-100 beats per minute
Temperature	98.2 degrees Fahrenheit	97-99 degrees Fahrenheit
BMI	19.85 kg/m^2^	18.5 - 24.9 kg/m^2^

**Table 2:** Serum laboratory studies with non-diagnostic results WBCs: White Blood Cells; ANA: Antinuclear Antibody; AB: Antibody; IFA: Immunofluorescence Assay; AST: Aspartate Aminotransferase; ALT: Alanine Aminotransferase

	Value	Reference Range
WBCs	8.4 x 10^9^ cells per Liter	4-10.5 x 10^9^ cells/L
Hemoglobin	13.1 g/dL	12-15 g/dL
Platelets	140,000 platelets/L	150,000-400,000 platelets/L
ANA Pattern	Nuclear Dense fines speckled	Negative
Anti-Nuclear AB by IFA (RDL)	Positive	Negative
Antinuclear Antibodies, IFA	1:320	Negative

**Figure 1:**
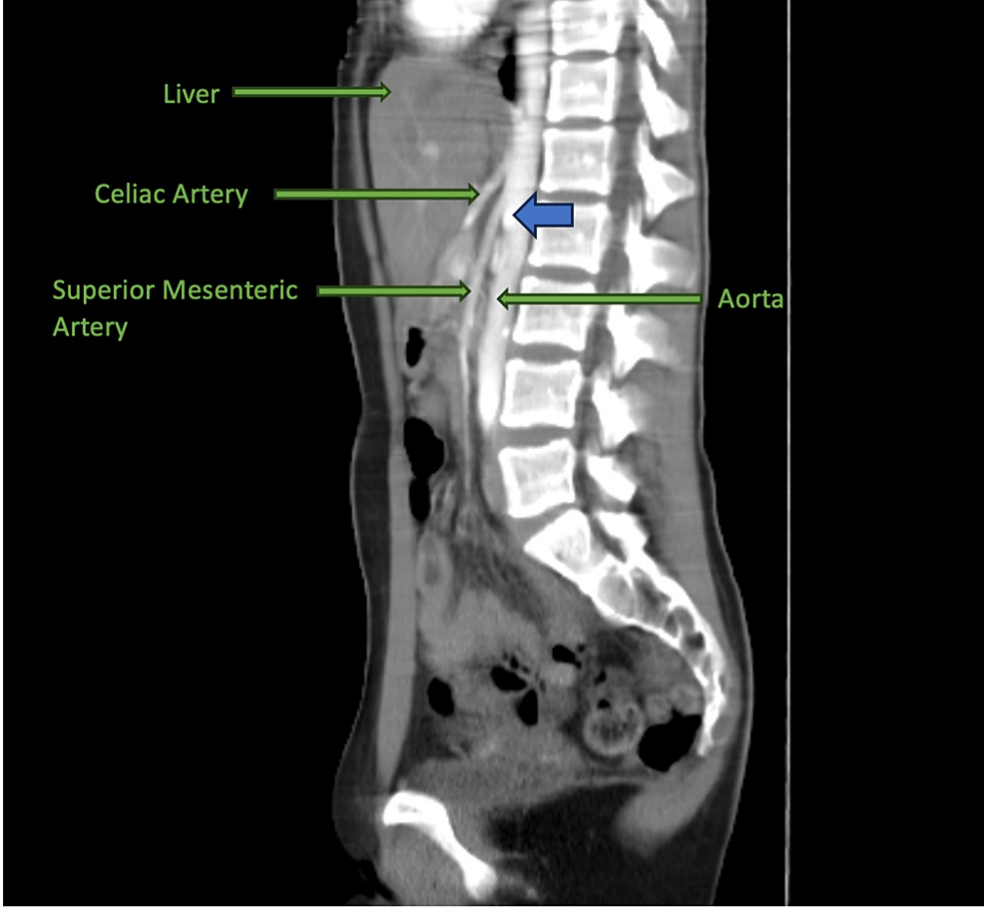
Compression of the duodenum between the root of the SMA and the abdominal aorta, labeled.

**Figure 2:**
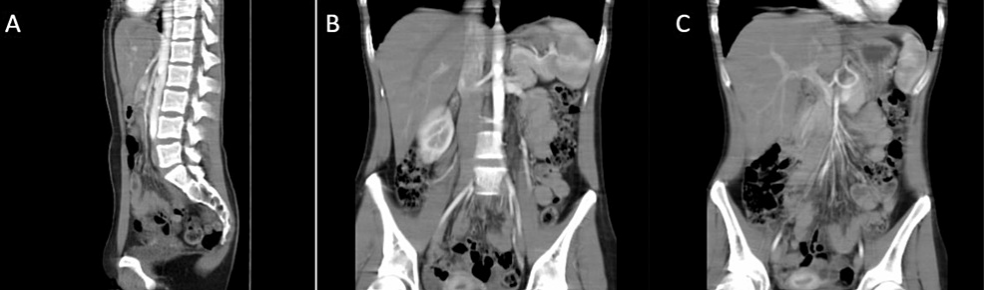
CT angiography of the abdomen and pelvis in multiple views. Lateral (A) and Coronal (B) views reveal an acute angle of the superior mesenteric artery together with compression of the second portion of the duodenum (C) by the superior mesenteric artery. A. CT angiography lateral view of the abdomen and pelvis revealing an acute angle of the superior mesenteric artery, as well as patent celiac axis, superior mesenteric artery, and inferior mesenteric artery B. CT angiography coronal view of the abdomen and pelvis revealing an acute angle of the superior mesenteric artery. C. CT angiography of the abdomen and pelvis showing compression of the second portion of the duodenum by the superior mesenteric artery.

A CT angiography of the abdomen and pelvis revealed an acute angle of the superior mesenteric artery with compression of the second portion of the duodenum (Figure 1, 2). The Celiac axis, superior mesenteric, and inferior mesenteric artery were all patent. The clinical signs and symptoms with investigative findings suggested the diagnosis of Superior Mesenteric Artery Syndrome, and the patient was referred to vascular surgery.

At the most recent three-month follow-up visit, the patient had a 10-pound weight gain and reported decreased intensity of her abdominal pain. Given her weight gain and commitment to nutritional support, and in concert with consultation with vascular surgery, surgical management was not deemed necessary. The patient was advised to continue small frequent meals with the goal of maintenance or additional weight gain to alleviate symptoms further.

## Discussion

SMA Syndrome is an uncommon cause of small bowel obstruction caused by compression of the duodenum by the SMA [3]. It was first described in a case report by Carl Van Rokitanski in 1842. Also known as Wilkie syndrome, chronic duodenal ileus, arterio-mesenteric duodenal compression syndrome, and cast syndrome, as little as 500 cases have been described in the literature since 2015 [2,3]. Although true incidence is unknown, commonly reported ranges are between 0.1-0.3% [4, 5]. Due to the rarity of SMA Syndrome, it is imperative to understand better the risk factors and clinical presentation to facilitate quicker diagnostics and referral for definitive management.

Common clinical presentation includes long-standing nausea, vomiting, and postprandial, intermittent, or chronic abdominal pain depending on the degree of obstruction [1-3]. Other presenting symptoms may include early satiety, weight loss, and abdominal distention. Prone or left lateral decubitus knee-chest position commonly offers relief due to normalization of the AO. This knee-chest positioning may explain why the patient presented in this case found some alleviation of symptoms with defecation. Regardless of the etiology, reducing the angle between the SMA and aorta can lead to partial or complete bowel obstruction [2, 5].

Diagnosis requires imaging to evaluate the AO, most commonly via CT angiography. In a case series of 8 patients, an AO angle of less than 22 degrees was seen to have 100% specificity and 42.8% sensitivity, and a less than or equal to 8 mm distance between the aorta and SMA showed both 100% specificity and sensitivity [6]. Other common imaging findings on plain radiographs include dilated stomach and diminished distal bowel gas. Endoscopy is often nonspecific but can be used to show complications, including gastric stasis, biliary reflux, gastritis, and duodenal ulcers. It may also help rule out other possible causes of duodenal compression with these nonspecific symptoms. Laboratory tests are usually nondiagnostic, as seen in this patient, with normalization of electrolytes, protein, and albumin in its chronic presentation [2].

Conservative medical management is typically the initial treatment for SMA syndrome. Small meals and posture therapy employing the left lateral decubitus can also improve symptoms. Nutritional support is equally crucial to improving symptoms by increasing the mesenteric fat pad and AO angle. Surgical intervention is typically required when conservative management fails, as evidenced by the persistence of pain and/or weight loss, or when complicated by pathology such as complete bowel obstruction, marked duodenal dilation, and complicating peptic ulcer disease. Many options exist for the surgical management of SMA Syndrome. These include Gastrojejunostomy, Strong’s Procedure-- division of the ligament of Treitz, transabdominal or laparoscopic duodenojejunostomy. Of these, the most commonly performed laparoscopic duodenojejunostomy; however, patient anatomy and surgeon preference may indicate using an alternative method [1, 2, 5]. Further, more recent reports have shown success in managing SMA syndrome via a newer method: infrarenal transposition of SMA [7, 8].

Diagnosis is often delayed, given an insidious onset of nonspecific symptoms, but morbidity and mortality remain high from complications. In patients diagnosed promptly, the prognosis is often positive, and patients respond well to conservative management. However, given the clinical presentation of nonspecific symptoms, SMA syndrome is often misdiagnosed as irritable bowel syndrome, gastroenteritis, or peptic ulcer disease. Conservative management for these disorders, including antiemetics, prokinetic agents, and proton pump inhibitors, is ineffective at relieving symptoms, and patients tend to worsen progressively over time. Resultantly, a cyclical pattern of worsening abdominal pain leads to further reduced appetite and greater degrees of intestinal obstruction, which progress to acute complications including: malnutrition, dehydration, electrolyte abnormalities, gastric pneumatosis and portal venous gas, gastrointestinal hemorrhage, small bowel obstruction, and gastric or intestinal perforation [2]. As seen in our patient, prompt recognition was missed for the initial differential diagnosis of allergies versus food sensitivities versus GERD. Thus, SMA syndrome should be considered in patients with appropriate risk factors and no clear etiology of abdominal pain. Of note, postprandial abdominal pain in the setting of anorexia or bulimia nervosa should raise particular suspicion for SMA syndrome, given the slightly higher prevalence in these populations. In our patient who fits the demographic mold, despite BMI being in the low-normal range, nonspecific abdominal symptoms, and nondiagnostic laboratory tests, SMA Syndrome remained a diagnosis of exclusion. Prompt confirmatory testing with CT Angiography allowed for recognition and surgical referral for therapeutic management.

## Conclusions

Superior Mesenteric Artery Syndrome is a rare upper gastrointestinal problem presenting nonspecific abdominal findings characterized by a narrowing angle between the aorta and SMA. A high clinical index of suspicion is required given the nonspecific presentation, which mimics peptic ulcer disease and irritable bowel syndrome. SMA Syndrome should be considered in a young female patient with low BMI and chronic, intractable postprandial abdominal pain unrelieved with standard management of more common conditions. Patients may present with unremarkable lab studies prompting the need for imaging studies, including CT angiography revealing an acute angle of the aortomesenteric angle. Due to its rare incidence, SMA syndrome should remain on the differential as a diagnosis of exclusion following similarly presenting symptoms for quicker diagnostics and referral for definitive management.
